# Implementation of Pharmacogenomics Testing in Daily Clinical Practice: Perspectives of Prescribers from Two Canadian Armed Forces Medical Clinics

**DOI:** 10.3390/jpm15030101

**Published:** 2025-03-04

**Authors:** Alexandra Muller-Gass, Gouri Mukerjee, Ruslan Dorfman, Rakesh Jetly

**Affiliations:** 1 Canadian Forces Health Services, Directorate of Mental Health, 60 Moodie Drive, Nepean, ON K1A 0K2, Canada; rakesh@drjetly.com; 2 Defence Research and Development Canada, Toronto Research Centre, 1133 Sheppard Ave. West, Toronto, ON M3K 2C9, Canada; 3 GeneYouIn Inc., 5000 Yonge St., Toronto, ON M2N 7E9, Canada; gourimukerjee@gmail.com (G.M.); ruslan@pillcheck.ca (R.D.); 4 Department of Anesthesia, McMaster University, Hamilton, ON L8S 4L8, Canada; 5 Institute of Mental Health Research, University of Ottawa, Ottawa, ON K1N 6N5, Canada

**Keywords:** pharmacogenomics, pharmacogenetics, personalized medicine, primary healthcare, psychiatry, military health services, implementation of pharmacogenomics, clinical decision support

## Abstract

**Background/Objectives**: While there is mounting scientific evidence supporting the effectiveness of PGx (pharmacogenomics)-guided medical treatment, its implementation into clinical care is still lagging. Stakeholder buy-in, in particular from prescribers, will be key in the implementation efforts. Previous implementation studies have primarily focused on prescriber attitudes or have used hypothetical scenario methodology in a variety of healthcare settings. Real-world studies provide better insight into prescriber experience and needs. In this prospective observational qualitative research study, we report the perspectives of prescribers working in military medical care after a one-year PGx implementation trial. **Methods**: At the end of the PGx implementation period, thirteen prescribers participated in a semi-structured interview. The interview was designed based on the Technology Acceptance Model and queried their perceptions of effectiveness and ease of use of the PGx innovation. **Results**: Three main themes emerged from the qualitative data: (1) the knowledge required for PGx testing, (2) the integration of the testing into the existing workflow and (3) the perceived clinical utility of the PGx results. Prescribers had educational and training opportunities prior to the study but still encountered difficulty with the interpretation of the test results. They generally managed well the workflow changes occasioned by the testing. They reported that the clinical value came primarily from an increased confidence in prescribing safe medications and improving the therapeutic alliance with their patients. There was uncertainty about which patient population would most benefit from the testing. **Conclusions**: Our results lend support to the general ongoing challenges identified in PGx implementation studies conducted in other clinical settings and using other methodologies. They also revealed specific factors that the prescribers found of value and areas that needed improvement to support future implementation efforts.

## 1. Introduction

The prevalence of major depression in Canadian Armed Forces (CAF) personnel is at about twice the levels seen in comparable civilian populations [1]. Hence, finding better strategies to prevent and treat mental health disorders is of high priority for military organizations. The current standard of care for mental disorders is based on “trial-and-error” until an effective treatment is identified; this approach can prolong illness and disability as well as frustrate both patient and clinician. Can we find ways to predict positive responses or at least narrow the choices of safe and effective medications?

One potential contributor to the field of personalized medicine is pharmacogenomics (PGx) testing, which identifies variations in the genes that can affect the way an individual processes or responds to some medications. Although genetic variation does not account for all the variability in treatment response and adverse side effects, the goal of PGx testing is to improve the drug efficacy and safety for each individual. PGx tests have been commercially available for close to two decades. Many studies, including randomized clinical trials, have demonstrated that PGx-guided treatment can improve clinical outcomes for some drugs (for recent reviews, see [2,3,4]). Because of its suboptimal drug therapy, studies have focused extensively on the field of mental health; however, PGx testing has also seen success in other therapeutic areas, including oncology, hematology, cardiology and pain management [5,6]. Regulatory agencies, such as Health Canada or the US Food and Drug Administration (FDA), have included PGx information on the label of over 100 medications, making it available for prescribers and patients. Moreover, several international consortia, for instance the Clinical Pharmacogenetics Implementation Consortium (CPIC) and the Canadian Pharmacogenomics Network for Drug Safety, have been established with the aim of developing clinical practice guidelines and to provide recommendations to prescribers.

The demonstration of various aspects of PGx testing has evolved substantially over the past decade [7]; however, the User Acceptance and Implementation Models domains remain an area of active research and investigation. Despite the growing scientific evidence, the use of PGx testing in routine healthcare remains limited and is primarily seen in large academic medical centers. One important consideration for its widespread adoption is the readiness of the end-users, the prescribers, to accept and employ PGx testing as a decision-support tool. Many previous studies have focused on prescriber attitudes, showing that prescribers generally have a positive attitude toward PGx testing but feel ill-equipped to use the test [8,9,10]. Real-world or hypothetical-scenarios studies that assess the impact of PGx testing on prescribers provide better insight into their experience and needs. More such studies are needed to reveal important prescriber-level challenges to the implementation of PGx testing, and this, in a variety of healthcare settings [11].

The purpose of the current study was to better understand the clinician’s experience with PGx testing in a military setting. What are the challenges and benefits perceived by the prescribers following a one-year PGx implementation period? Interview questions and response analysis were guided by an adaptation of the Technology Acceptance Model (TAM). The TAM is a theoretical model proposed to assess end users’ acceptance behavior toward technology; it has been tested over a spectrum of applications, and studies have provided evidence that TAM is a good predictor of behavioral intent to accept technology in the health sector [12]. In TAM, technology acceptance is strongly affected by the ‘perceived ease of use’ and the ‘perceived usefulness’ of the technology [13].

## 2. Materials and Methods

### 2.1. Participants

Forty-four prescribers participated in the implementation study. They were employed at one of two study sites: Ontario (2 Field Ambulance Petawawa) or Atlantic (Canadian Forces Health Services). Both sites provide outpatient medical care, including mental health services. Inclusion in the interview phase of the study required that prescribers had ordered a minimum of ten PGx tests by the end of the implementation period. This criterion ensured that the prescriber had enough experience with the PGx testing process and could provide an informed opinion on acceptance and perceived clinical value.

This research was approved by the Human Research Ethics Committee (HREC 2017-058 15 February 2018) at Defense Research and Development Canada according to Canada’s Tri-Council policies and guidelines for research involving human subjects. All participants provided signed consents prior to the start of the study. They did not receive compensation for their time.

### 2.2. Procedures

Prior to the start of PGx implementation, all prescribers were offered several education strategies: (i) in-person workshops (30–40 min) with a senior CAF healthcare expert and PGx expert in attendance; (ii) printed educational materials designed to help prescribers explain the concept of PGx to patients; (iii) five webinars (self-learning) of 20–30 min each that explained PGx science and its applications to different medical areas; and (iv) a personal PGx test.

The implementation of PGx testing was performed from March 2018 until February 2019. Prescribers introduced patients to the PGx study and gave a requisition slip to the patients interested in PGx testing. A support staff member was responsible for the local logistics and coordination of the PGx study. They obtained written consent from the patients, collected the sample (cheek swab), prepared it for shipping, received the test results and ensured that the PGx result reports were included in the patients’ EMR. Prescribers were informed by email when results were scanned into the patients’ EMR. At the Atlantic clinic, prescribers could consult with the onsite clinical pharmacist (CP) for support with the interpretation of the test results. The CP was debriefed about their experience at the end of the study.

The interview phase of the study used a qualitative descriptive approach with a constructivist research paradigm and a purposeful sampling technique. Prescribers meeting the inclusion criterion were contacted directly by email or in person by the support staff to request their participation in the interview. The individual interviews were conducted in early 2019 by two authors (RD and GM or RJ and GM) and were audio recorded.

### 2.3. Interviews

The semi-structured interview included open-ended questions developed to encourage detailed and personalized responses (see Table S1). The research team developed the interview instrument through discussion and iterative refinement. Interview questions were divided into categories: (1) role, years of practice and familiarity with PGx, (2) usefulness of PGx test, (3) ease of ordering tests, receiving results, interpreting tests, and clarity of the result reports, (4) adequacy of educational materials, and (5) open-ended feedback. The interview prompted respondents to discuss the use of the PGx tool in the context of their practice as it related to their experiences with individual patients.

### 2.4. Data Analysis

The audio interview files were transcribed manually and two authors (RD and GM), working independently, reviewed the transcripts for accuracy. Two authors (AMG and GM) coded five random interview transcripts independently to identify predominant themes in the data. The codebook was refined through discussion (RJ, AMG and GM), and consensus was obtained on the organization of relevant subtopics within the larger themes. Subsequently, AMG and GM separately analyzed each interview according to the codebook and mapped back the results to the TAM concepts of ease of use and usefulness.

### 2.5. PGx Test Report

PGx testing included a panel of 18 genes and 96 markers for 179 drugs irrespective of the specific drug that the prescriber was interested in at the time of ordering (see Figure S1). Results were presented in two formats: (1) a PGx summary table in which drugs were categorized as ‘use as recommended’, ‘use with caution’ or ‘use with increased caution and monitoring’ and (2) a 3–4 page PGx short report with medications categorized into three color-coded sections indicating risk of gene–drug interactions by therapeutic area, identified diplotype (e.g., CYP2D6 *1/*2) and functional phenotype (Poor, Intermediate, Normal and Rapid Metabolizer) (see Figure S2). The PGx summary table and short report were scanned into the EMR of patients. A more in-depth PGx report was made available to prescribers if requested.

## 3. Results

### 3.1. Interview Participants

Thirteen of the 44 prescribers met the criterion to proceed to the interview phase. Most of these prescribers were psychiatrists (*n* = 7); they also included a dental surgeon, a family physician, and four nurse practitioners. Most prescribers worked at their respective bases full-time (*n* = 12), and their years of professional experience ranged from 1 to 23 years (average = 5.5). Five of the prescribers worked at the Ontario clinic and 8 at the Atlantic clinic.

### 3.2. Perceived Ease of Use

#### 3.2.1. Knowledge and Training Methods

All psychiatrists had previous knowledge of PGx technology, and two of them had practical experience. The other prescribers were not aware of this technology before the study. All prescribers agreed on the necessity of training to maximize the benefits of PGx technology. Several psychiatrists mentioned that the training would especially benefit family physicians and nurse practitioners as they would have had little exposure to this technology in their formal education. In terms of training formats, most prescribers considered the in-person workshops most useful, and a few wished it had been more interactive. Many clinicians appreciated the convenience of webinars that could be accessed according to their personal work schedules. The time required for training was frequently mentioned as a drawback. A few clinicians could not complete all the offered training. All clinicians felt adequately prepared to use PGx following the training. A few clinicians mentioned that more training should be provided for the interpretation of the results. One prescriber suggested that the training could include reviewing case studies using PGx among colleagues:

*“I think more educational sessions. More sitting down with colleagues, sharing your experiences, sharing examples. Grand rounds, clinical scenarios. I think that will stick with us. If you could do a 1-day training on how to interpret results, very useful”.*

As part of training, most prescribers completed a personal PGx test and found that this exercise helped to “*understand the process, see results, understand value, explain to patients*”. One prescriber refused the personal PGx test out of concerns for privacy.

#### 3.2.2. Effects on Workflow

All prescribers agreed that introducing the PGx study to patients was simple and took less than 5 min. Some prescribers handed over a PGx pamphlet developed for patient education, while others developed their own short pitch to introduce PGx. In general, prescribers felt that patients would not benefit from more extensive education about PGx. One prescriber mentioned that prescribers should emphasize that the purpose of the test was not to find a medication that has therapeutic effectiveness, which is a common misinterpretation of the patients. Most patients were keen and agreed to the test; it was estimated that about 10% declined, which was mainly because of concerns about privacy related to genetic testing. Almost all prescribers found the test ordering easy, taking only a few minutes. Two prescribers suggested that this ease was due to the support staff helping with test ordering.

Most prescribers found that the results were received in an acceptable timeframe (typically 1–2 weeks). Many did not delay their clinical decisions but rather adjusted treatment as needed following the receipt of results. A few prescribers sometimes waited (e.g., in complex cases) for the PGx results before commencing treatment. There was consensus that the PGx summary table presented the results in a straightforward manner and provided sufficient information. Some said that reading the short report was too time consuming. The issue of time constraints led to the suggestions of providing bullet point results, highlighting relevant information in the PGx report and providing prescription alerts in the EMR. One prescriber suggested the following:

*‘One thing that might be useful, having something next to the drug in the list, putting something like beyond “use with increased caution”, like “should absolutely not be used”, or “consider dose reduction”, or “consider dose increase”(…) you might want to break these things down more in terms of severity and what to do with them”.*

When results were normal, the interpretation of the results was quick and easy. However, in cases of altered metabolism, especially when the case was complex (e.g., polypharmacy), they found the interpretation of results the most time consuming and complicated step in the PGx implementation. When difficulties with the interpretation arose, several prescribers (especially primary care) from the Atlantic clinic consulted with the CP. One prescriber said the following:

*“I didn’t feel comfortable making big decisions with these summaries. From a basic, family doctor, general practitioner point of view, this is a test that needs to be reviewed with a pharmacist or with someone else that can help”.*

Almost all prescribers reported that the communication of test results to patients was straightforward and took less than 5 min. Some prescribers found it helpful to show patients their summary report with color-coded results or let them review their own report before the appointment. A few prescribers preferred routinely referring their patients to the CP to help explain the results. In their debrief, the CP mentioned that the full PGx report with in-depth information for each patient helped them understand and explain to the patients why a particular drug was recommended for them.

### 3.3. Perceived Usefulness

#### 3.3.1. Support to Clinical Decision-Making

Most prescribers trusted the results of the PGx test report and perceived them as useful in supporting clinical decision making. In most cases, the results of the PGx test were normal and therefore did not affect clinical management. Still, prescribers found value in knowing this information, and it increased their confidence in prescribing medications. In the other cases, results showed altered metabolizers and were taken into consideration to guide clinical decisions. Several prescribers reported that they only had a few patients with altered drug metabolism, while two prescribers estimated that up to 30% of cases had altered metabolism. Most prescribers believed that the PGx testing improved clinical outcomes for these patients. Three prescribers, however, mentioned that some of their patients had reacted well to medications that were labeled “use with caution” or contraindicated in the PGx test report. One of these prescribed reported the following:

*“(…) there were a couple of times where I had a couple of clients who might’ve been on, say, an antidepressant that were in the area, “Use with caution”, but were doing fine. And again, we would have a discussion about that”.*

One prescriber believed that the PGx test results explained some of their non-responder patients. Another prescriber reported that the PGx test results were compatible with the prior negative clinical experience of a patient who was a slow metabolizer; they thought that PGx testing could have prevented the severe side effects experienced by the patient:

*“(…) every starting medication there were bad side effects, severe side effects. It didn’t matter what medication I gave him or how low a dose I got, there were still side effects (…) And in my mind, I was thinking, “Maybe this fellow is a slow metabolizer, so he’s very sensitive to the side effects.” I did PGx testing on him, and it really just confirmed that. It was confirmatory for what I experienced clinically”.*

#### 3.3.2. Patient Engagement

Most prescribers reported that an unexpected effect of the PGx trial was an improved therapeutic alliance between patient and prescriber. One prescriber mentioned that they did not always see an improved clinical outcome but saw much-improved patient engagement; it made patients feel part of the process. Another prescriber reported that the best value of the PGx trial was patient buy-in and that the PGx summary report was a great patient interaction tool. Two prescribers mentioned that patient buy-in may lead to a placebo effect that could partly explain improved clinical outcomes in other studies.

All prescribers felt that the PGx test results increased, in a subset of the patient population, acceptance, adherence, compliance and/or patient confidence with the prescribed medication. Several prescribers reported that it helped convince some reluctant patients to try a new medication, whereas it reassured other patients that the medication was compatible with them. One prescriber reported the following:

*“Patient with treatment resistant depression for a long period of time. More than 7 or 8 medication trials that he hasn’t responded to very well [maximum 15% improvement] (…) He was failing to trust myself and the other treatment providers (…) [After the PGx testing] I can see with this medication trial, he’s at least having 30% of the symptom benefit and I can see him engaging into other psychosocial interventions”.*

#### 3.3.3. Key Test Population and Medical System Efficiencies

Some prescribers used the test frequently on all patients requiring medication; one prescriber even ordered the test pre-emptively in case patients would need medication in future. These prescribers did not feel that there was a risk associated with PGx testing and applied it to a broad population. Other prescribers were more selective in choosing their test population. Many believed that it should be a routine test for mental health patients or those taking multiple medications. Others suggested it should be performed on treatment-resistant patients or those experiencing many side effects. One prescriber found it helpful before planned surgeries to guide post-op pain management and suggested it could be used if there is suspicion of drug-seeking behavior for more potent narcotics. Another prescriber said that the PGx results could serve as support to obtain special authorization to take an alternative medication that is covered only in special circumstances.

Most prescribers, especially psychiatrists, agreed that they should have PGx results in advance of a consult before starting patients on a new medication. One psychiatrist was pleased to receive the PGx report from a primary care prescriber who had ordered it. Several prescribers suggested that the results should be on file for all prescribers to share. Several prescribers said that it could help primary care prescribers perform their job more quickly and safely as they often work outside their area of comfort; it could also lead to fewer mental health referrals. Most prescribers also found it important to share the PGx report with the patients for future care. Some informed patients when medications were flagged on the report due to altered metabolism even when not relevant to the current treatment. Others gave patients a copy of their results to self-manage or to take the report to other prescribers providing care.

## 4. Discussion

The objective of this study was to better understand the views of prescribers about using PGx testing in their daily practice. While there are several defense research programs and studies that have used pharmacogenetics to improve military clinical care [14,15], to our knowledge, this is the first implementation study in which PGx testing was introduced for routine use by prescribers in military clinics. Many of our findings overlapped those reported by PGx studies carried out in other contexts of care and services [16]. The prescribers participating in our study shared the benefits and challenges experienced with PGx testing along three main themes: (1) the knowledge required for PGx testing, (2) the integration of the testing into the existing workflow, and (3) the perceived clinical utility of the PGx results.

### 4.1. Knowledge Required for PGx Testing

The knowledge gap remains the most pervasive barrier to PGx implementation [17,18]. The prescribers’ lack of education about PGx affects their decision to order initial testing as well as their ability to interpret the results and counsel their patients [18,19,20]. We offered our prescribers a variety of PGx learning opportunities to encourage test usage by making them more familiar with PGx procedures and science. Consistent with other studies, prescribers preferred the in-person workshops [21,22,23,24,25] and found that personal PGx testing gave them insight into the patient experience. Personal PGx testing as a training method has shown to improve the understanding of genetic concepts and impact perceptions of clinical utility [26,27,28,29,30]. All participants felt adequately prepared to use PGx testing following our training. Despite this, only 13 of the 44 prescribers ordered tests for at least ten patients during the implementation period. Studies have reported that training increased confidence in ordering and interpreting the PGx test but did not increase the number of PGx tests ordered during their study [23,31,32]. In our study, prescribers struggled most with translating the PGx results into clinical management. This highlights the importance of ensuring the PGx education includes the interpretation of the results not just the scientific basis of PGx.

### 4.2. Integrating PGx into the Existing Clinical Workflow

Some studies have suggested that the current PGx testing process may be too cumbersome and lengthy to be easily included into the prescriber’s existing workflow [19,26]. Our results indicate that the prescribers faced few challenges with workflow changes, although they were supported by dedicated staff during the study. The prescribers considered the interpretation of the PGx results to be the most time-consuming step, especially in polypharmacy cases. Some providers appreciated the support of a clinical pharmacist for this purpose. The involvement of the clinical pharmacist in the implementation of PGx has received strong support from medical practitioners and leading professional organizations [11,24,32]. The interpretation of PGx results can also be facilitated by a clear result report [26]. In our study, most prescribers preferred receiving the results in the short summary table. They suggested to highlight the most pertinent information to make the results even more understandable.

In a recent review paper, the relatively long time required to receive results is also discussed as an important barrier to PGx implementation [33]. Most of our prescribers did not consider turnaround time (approx. 1–2 weeks) as a major obstacle. Few reported having delayed their clinical decisions until receipt of the results. While turnaround time may dissuade prescribers from ordering a PGx test, once the PGx results are added to the patient’s EMR, it no longer remains an obstacle for subsequent prescription decisions for that patient.

### 4.3. Clinical Utility of the PGx Results

Evidence for the clinical utility of PGx testing remains a major barrier to its adoption, and this despite supportive results from several RCTs [34,35]. The concept of clinical utility has no fixed definition and can include different aspects and endpoints based on the perspective of the particular stakeholder. In our study, prescribers assessed clinical utility in relation to their prescription decisions but also discussed other value factors. Many prescribers thought that PGx testing had helped with their medication choice for patients with altered drug metabolism, and some believed it explained past medication experiences. In general, even if the PGx test did not often change clinical management, prescribers found it to be valuable to validate and increase confidence in their medication decisions. Furthermore, many prescribers were surprised by the overwhelmingly positive effect of the PGx testing on their patients. As reported in other studies, our prescribers described that it improved patient engagement, communication and confidence in the proposed treatment [33]. Regardless of evidence of clinical utility, using PGx seemed to convey a sense of rational psychopharmacology rather than the typical “trial-and-error approach”. Interestingly, two prescribers mentioned that the patient buy-in effect may be a confounding variable and could account for some of the improved clinical outcomes in other studies.

There was a good deal of variability with respect to the context in which prescribers chose to administer PGx testing. Differing perceptions of which patients are likely to benefit from PGx testing is frequently reported in other studies [11,36]. Some prescribers tested routinely or pre-emptively, while others were more selective and used it only with certain patient populations. A recent review suggests that preemptive PGx testing could significantly reduce healthcare resource utilization by predicting and preventing adverse drug reactions [37]. PGx implementation has shown clinical value in both primary and secondary care settings [33]. Primary care providers will be important PGx stakeholders because they care for many patients that could become PGx users. In our study, several prescribers commented on the value of PGx to primary care practitioners who often work outside their area of expertise. They thought it would help them work faster, more safely, and potentially reduce the need for psychiatric consultation.

### 4.4. Limitations

There are several limitations in our study, especially related to participant selection, that require consideration when interpreting the results. Military medical clinics have unique demands, patients and workflows that could have affected the prescribers’ experiences with PGx testing. While our results are similar to those found in studies conducted in other medical contexts, it nevertheless does limit the transferability to civilian healthcare settings. In qualitative studies, there are often restrictions in sampling strategies, and this can affect the sample size and generalizability of results. A positive sampling bias could have been introduced in that the prescribers who are more receptive or experienced with PGx testing may have been more likely to volunteer for the study and meet the study inclusion criterion. Therefore, the perspectives presented in this report may not be fully generalizable to the opinions within the larger CAF prescriber population. Another limitation in this study is that participants who did not meet the inclusion criterion, which accounted for 31 of the initial 44 participants, were not queried about the reasons for their infrequent use of the tests. This information could have provided valuable insight into the challenges of PGx testing. With only about 20% of patients having actionable PGx results, these prescribers may have not experienced enough early benefit to continue ordering the tests. Surveys of prescribers have shown that they often experience enthusiasm for PGx testing, but this did not translate into ordering more tests [38,39]. These results highlight the need for studies to better understand the factors affecting the prescriber uptake of pharmacogenomic (PGx) testing to avoid unequal patient access. Ensuring equitable access to PGx testing depends on both prescriber and patient considerations and remains an active area of implementation research [40,41].

## 5. Conclusions

The results of the present study contribute to a better understanding of the real-world experiences of the main PGx stakeholders, the prescribers. It also provides actionable insights and efficiencies for the introduction of PGx in military clinical care. The barriers discussed by the providers from the CAF medical clinics are similar to those found in other clinical settings: namely, a deficit of knowledge about PGx, workflow issues and uncertainty about the clinical utility of the test. Mitigating strategies could include a pragmatic-focused educational curriculum, well-defined procedures and concise formats for the reporting of results, a better delineation of the target patient population, and the provision of human resources support. In addition to demonstrating the clinical utility of PGx testing, further research should focus on other important aspects for the implementation of PGx, such as cost-effectiveness, accessibility, confidentiality and related ethical issues.

## Data Availability

The data presented in this study are available on request from the corresponding author because they contain information that could compromise the privacy of the study participants.

## Supplementary Materials

The following supporting information can be downloaded at https://www.mdpi.com/article/10.3390/jpm15030101/s1, Table S1: Interview guide with open-ended questions and probes, Figure S1: List of genes and alleles included in the PGx testing, Figure S2: Example of a PGx short report.

## Abbreviations

The following abbreviations are used in this manuscript:

PGx	pharmacogenomics
CAF	Canadian Armed Forces
TAM	Technology Acceptance Model
CP	clinical pharmacist
