# Supplementary material for: Implementation of Pharmacogenomics Testing in Daily Clinical Practice: Perspectives of Prescribers from Two Canadian Armed Forces Medical Clinics
Source: J Pers Med. 2025 Mar 4;15(3):101. doi: 10.3390/jpm15030101 (PMC11943113; doi:10.3390/jpm15030101)
Supplement: Supplementary file 1 [file jpm-15-00101-s001.zip › Figure S1.pdf]

**Figure S1:** List of genes and alleles included in the PGx testing

| Gene | Alleles tested |
|---|---|
| ADRB2 | rs1042713 G/A |
| CYP1A2 | *1E, *1F, *1J, *1K, *6, *7, *8, *15 |
| CYP2B6 | *2, *5, *6, *7, *8, *13, *18, *22, *34 |
| CYP2C19 | *2, *3, *4, *6, *8, *10, *17 |
| CYP2C8 | *2, *3, *4 |
| CYP2C9 | *2, *3, *8, *9, *11, *12, *27 |
| CYP2D6 | *3, *4, *5, *6, *7, *10, *17, *29, *41, *64, *69, *82 and CNV |
| CYP3A4 | *3, *6, *11, *12, *16, *17, *18, *19, *22 |
| CYP3A5 | *2, *3, *6 |
| DYPD | *2A, *4, *5, *6, *7, *8, *9A, *9B, *10, *13, rs67376798A |
| F2 | 20210G>A (rs1799963) |
| F5 | Factor V Leiden (rs6025) |
| IFNL3 | rs12979860 C/T |
| OPRM1 | A118G (rs1799971) |
| SLCO1B1 | *2, *3, *5, *6, *9, *10, *11, *12, *13, *15, *31 |
| TPMT | *2, *3A, *3B, *3C, *4, *8 |
| UGT1A1 | *6, *7, *27, *29, *60 |
| UGT2B15 | rs1902023 A/C |
| VKORC1 | c.-1639G>T (rs9923231) |

**Technology:** Genotyping was performed using the Applied Biosystems™ QuantStudio™ platform, and this report is powered by Pillcheck technology.

**Limitations:** This test will not detect all known mutations that result in altered gene activity. *1 or wild-type alleles are reported by default if those listed were not detected. IND values are conservatively assigned to alleles that could not be determined with complete certainty. Only listed mutations are tested for and absence of a detected mutation does not rule out the possibility of sensitivity to a specific drug due to the presence of other mutations or other environmental factors.

Additional genetic testing by sequencing might uncover other functional variations that the individual may carry that also affect the medication response but were not detected in this analysis.
