# Supplementary material for: Implementation of Pharmacogenomics Testing in Daily Clinical Practice: Perspectives of Prescribers from Two Canadian Armed Forces Medical Clinics
Source: J Pers Med. 2025 Mar 4;15(3):101. doi: 10.3390/jpm15030101 (PMC11943113; doi:10.3390/jpm15030101)
Supplement: Supplementary file 1 [file jpm-15-00101-s001.zip › Figure S2.pdf]

Patient Code: [numerical code]
Sample Barcode: [numerical code]
Date Prepared: [DATE of report]
Referring Clinician: Prescriber's name

pillcheck

Pillcheck Pharmacogenetics (PGx) Summary

This is a limited summary of Pillcheck pharmacogenetics test results for requested therapeutic areas. Patient's complete Pillcheck Report covering detailed recommendations for over 148 medications and additional therapeutic areas is available on the Pillcheck Portal. To review full report please log into your account: https://portal.pillcheck.net. Contact [project coordinator's email] for assistance or to request consultation with a PGx-trained clinical pharmacist.

Treatment Areas

Medicine	Use with increased caution - consider alternatives	Use with caution - more frequent monitoring	Use as directed - standard precaution
Analgesics	Codeine<br>Hydrocodone<br>Oxycodone<br>Tramadol and acetaminophen	Ketamine<br>Methadone	Celecoxib<br>Diclofenac<br>Fentanyl<br>Flurbiprofen<br>Hydromorphone<br>Morphine<br>Naloxone<br>Naltrexone<br>Piroxicam
Antibacterial		Telithromycin	
Antiemetics		Aprepitant<br>Fosaprepitant	Dolasetron<br>Dronabinol<br>Ondansetron<br>Palonosetron<br>Tropisetron
Antifungals	Terbinafine	Itraconazole<br>Voriconazole	
Antiviral		Atazanavir<br>Dolutegravir<br>Efavirenz<br>Fosamprenavir<br>Nevirapine	Boceprevir<br>Daclatasvir<br>Elbasvir and grazoprevir<br>Ledipasvir and sofosbuvir<br>Ombitasvir, paritaprevir and ritonavir<br>Peginterferon Alpha-2b<br>Simeprevir<br>Sofosbuvir<br>Sofosbuvir and velpatasvir<br>Telaprevir

Medicine	Use with increased caution - consider alternatives	Use with caution - more frequent monitoring	Use as directed - standard precaution
Cardiovascular	Carvedilol<br>Clopidogrel<br>Flecainide<br>Metoprolol<br>Propafenone<br>Propranolol<br>Quinidine<br>Ranolazine<br>Timolol	Amlodipine<br>Atorvastatin<br>Dronedarone<br>Fluvastatin<br>Irbesartan<br>Lovastatin<br>Nebivolol<br>Pitavastatin<br>Pravastatin<br>Rosuvastatin<br>Sildenafil<br>Simvastatin<br>Tadalafil<br>Vardenafil	Alirocumab<br>Cilostazol<br>Evolocumab<br>Losartan<br>Prasugrel<br>Ticagrelor
Dermatology and Dental	Cevimeline		
Endocrinology	Eliglustat		
Gastroenterology			Dexlansoprazole<br>Esomeprazole<br>Lansoprazole<br>Omeprazole<br>Pantoprazole<br>Rabeprazole
Gynecology			Desogestrel<br>Ethinylestradiol
Hematology		Apixaban<br>Rivaroxaban	Acenocoumarol<br>Phenprocoumon<br>Warfarin
Immune therapy		Methotrexate	Cyclosporine<br>Sirolimus<br>Tacrolimus
Musculoskeletal		Carisoprodol	
Neurology	Dextromethorphan and quinidine<br>Donepezil<br>Galantamine<br>Tetrabenazine<br>Valbenazine	Brivaracetam<br>Caffeine<br>Clobazam<br>Deutetrabenazine<br>Lacosamide<br>Rasagiline	Fosphenytoin<br>Midazolam<br>Phenytoin<br>Valproic acid / divalproex

Patient Code: [numerical code]
Sample Barcode: [numerical code]
Date Prepared: [DATE of report]
Referring Clinician: Prescriber's name

Medicine	Use with increased caution - consider alternatives	Use with caution - more frequent monitoring	Use as directed - standard precaution
Oncology	Gefitinib<br>Tamoxifen	Belinostat<br>Cabazitaxel<br>Erlotinib<br>Irinotecan<br>Nilotinib<br>Pazopanib	Capecitabine<br>Cisplatin<br>Enzalutamide<br>Fluorouracil<br>Mercaptopurine<br>Tegafur<br>Thioguanine
Other		Flibanserin	
Psychiatry	Amitriptyline<br>Amphetamine<br>Aripiprazole<br>Atomoxetine<br>Brexpiprazole<br>Chlordiazepoxide and amitriptyline<br>Clomipramine<br>Clozapine<br>Desipramine<br>Doxepin<br>Duloxetine<br>Fluoxetine<br>Fluoxetine and olanzapine<br>Fluvoxamine<br>Haloperidol<br>Iloperidone<br>Imipramine<br>Mirtazapine<br>Modafinil<br>Nefazodone<br>Nortriptyline<br>Paroxetine<br>Perphenazine<br>Pimozide<br>Protriptyline<br>Risperidone<br>Thioridazine<br>Thiothixene<br>Trimipramine<br>Venlafaxine<br>Vortioxetine<br>Zuclopenthixol	Buprenorphine<br>Bupropion<br>Chlorpromazine<br>Clonazepam<br>Diazepam<br>Eszopiclone<br>Guanfacine<br>Lorazepam<br>Lurasidone<br>Olanzapine<br>Oxazepam<br>Trazodone<br>Zopiclone	Alprazolam<br>Citalopram<br>Escitalopram<br>Quetiapine<br>Sertraline
Pulmonary	Salmeterol	Indacaterol	

Patient Code: [numerical code]
Sample Barcode: [numerical code]
Date Prepared: [DATE of report]
Referring Clinician: Prescriber's name

pillcheck

**Medicine**	**Use with increased caution - consider alternatives**	**Use with caution - more frequent monitoring**	**Use as directed - standard precaution**
Rheumatology			Azathioprine<br>Lesinurad
Urology	Darifenacin<br>Fesoterodine<br>Tamsulosin<br>Tolterodine		

PGx Summary - Psychiatry

Biomarker	Value	Summary
CYP2D6	*4/*4 (2N)	Decreased metabolism of aripiprazole is anticipated, start at 50% of the usual starting dose and adjust to achieve favorable clinical response. Avoid using amitriptyline and other TCAs; if use is warranted, verify CYP2C19 metabolism and consider a 50% dose reduction. Consider using citalopram or sertraline as significant side effects to paroxetine, fluvoxamine and other CYP2D6-dependent SSRIs are anticipated. Reduced metabolism of venlafaxine can affect clinical response, select alternate drug if possible. Significantly decreased clearance of amphetamine, donepezil and clozapine may lead to higher risk of adverse reactions.
CYP2B6	*1/*2	Possible decreased metabolism of bupropion to hydroxybupropion, an active metabolite. Suggest to monitor efficacy and tolerability of bupropion.
CYP2C19	*1/*2	Reduced metabolism of citalopram, escitalopram and sertraline is anticipated which may lead to enhanced response. Initiate therapy with recommended starting dose and be alert to signs of side effects. Amitriptyline, doxepin and imipramine are partly metabolized by CYP2C19. Verify CYP2D6 metabolism, if CYP2D6 metabolism is normal, for CYP2C19 intermediate metabolizers, no empiric dose adjustments are recommended however suggest to monitor closely for side effects. Reduced metabolism of clobazam may lead to a higher risk for adverse effects, but also a better response to treatment. Consider starting at lower doses and proceed slowly with dose titration. Patients receiving diazepam for preoperative anxiety may have reduced clearance and may therefore take longer time to emerge from anaesthesia.
CYP3A4	*1/*22	Reduced metabolism of clonazepam, guanfacine, trazodone, eszopiclone, zopiclone is anticipated, increasing risk of side effects.
UGT2B15	CA	Reduced clearance of lorazepam and oxazepam is anticipated, consider starting at lower doses.
CYP1A2	*1F/*1F	Increased clearance of rasagiline, olanzapine and chlorpromazine, which may lead to reduced clinical response and potential need to increase dosage. Pregnant women with ultrarapid CYP1A2 metabolism who consume caffeine may have an increased likelihood of spontaneous abortion as compared to patients with reduced metabolism.
CYP2C9	*1/*1	Normal metabolism of phenytoin, valproic acid and divalproex is anticipated.
CYP3A5	*3A/*3A	The majority of Caucasians do not express the CYP3A5 enzyme. As such, poor CYP3A5 metabolizers are expected to have a normal clearance of quetiapine, alprazolam and midazolam.

PGx Summary - Cardiovascular

Biomarker	Value	Summary
CYP2D6	*4/*4 (2N)	Decreased metabolism of metoprolol, suggest alternative drug (e.g. bisoprolol) or reduce dose by 75%. For flecainide, reduce starting dose by 50%, whereas for propafenone, reduce starting dose by 70% or select alternative drug (sotalol, disopyramide, amiodarone). For timolol, be alert to potentiated systemic beta-blockade, as decreased drug clearance may result in increased risks of side effects.
CYP2C19	*1/*2	Decreased transformation of clopidogrel into its activate metabolite is possible, which may result in decreased efficacy. If antiplatelet therapy is required, suggest using prasugrel or ticagrelor instead of clopidogrel if clinically indicated.
CYP3A4	*1/*22	Reduced drug metabolism is anticipated for apixaban and rivaroxaban, which may lead to increased drug exposure and risk of bleeding. Reduced clearance of sildenafil, tadalafil, vardenafil and amlodipine may increase drug exposure and the risk of side effects. Reduced dronedarone clearance is anticipated increasing the risk of QT interval prolongation and induction of Torsade de Pointes.
SLCO1B1	*1A/*15 or *1B/*5	Higher plasma concentration of cholesterol-lowering agents, such as atorvastatin, fluvastatin, pravastatin, rosuvastatin and simvastatin is anticipated, which may lead to an increased risk of adverse effects, such as elevated creatinine kinase levels or myopathies, consider starting at a lower dose.
CYP2C9	*1/*1	Normal metabolism of acenocoumarol and warfarin is anticipated. For starting warfarin and acenocoumarol dose guidance and VKORC1 status refer to full PGx report. Normal metabolism of irbesartan and losartan is also anticipated.
VKORC1	GG	Normal Vitamin K levels. For starting warfarin and acenocoumarol dose guidance and CYP2C9 status refer to full PGx report.
CYP3A5	*3A/*3A	The majority Caucasians do not express the CYP3A5 enzyme. As such, poor CYP3A5 metabolizers are expected to have a normal response to cilostazol.

PGx Summary - Analgesics

Biomarker	Value	Summary
CYP2D6	*4/*4 (2N)	Avoid using amitriptyline and other TCAs; if use is warranted, verify CYP2C19 metabolism and consider a 50% dose reduction. Consider using citalopram or sertraline as significant side effects to paroxetine, fluvoxamine and other CYP2D6-dependent SSRIs are anticipated. Reduced metabolism of venlafaxine can affect clinical response, select alternate drug if possible. Avoid using codeine, oxycodone, tramadol, hydrocodone due to significantly reduced activation leading to reduced pain relief, suggest alternate analgesics such as morphine, hydromorphone or non-opioid drugs. See below for opioid response according to OPRM1 gene.
OPRM1	AA	Normal response to opioids is anticipated.

Patient Code: [numerical code]
Sample Barcode: [numerical code]
Date Prepared: [DATE of report]
Referring Clinician: Prescriber's name

Biomarker	Value	Summary
CYP2B6	*1/*2	Intermediate metabolizers may exhibit higher levels of methadone in their blood than normal. If use of methadone is required in the future, consider starting at low doses of methadone and increasing doses slowly. Reduced ketamine clearance is anticipated, suggest to monitor for increased side effects.
CYP2C19	*1/*2	Decreased metabolism of carisoprodol is anticipated, leading to increased risk of side effects. Use with caution and be alert to signs of side effects. Reduced metabolism of citalopram, escitalopram and sertraline is anticipated which may lead to enhanced response. Initiate therapy with recommended starting dose and be alert to signs of side effects. Amitriptyline, doxepin and imipramine are partly metabolized by CYP2C19. Verify CYP2D6 metabolism, if CYP2D6 metabolism is normal, for CYP2C19 intermediate metabolizers, no empiric dose adjustments are recommended however suggest to monitor closely for side effects. Reduced clearance of PPIs may result in increased exposure. No dose adjustments or increased monitoring required.
CYP3A4	*1/*22	Reduced buprenorphine metabolism may cause higher buprenorphine exposure increasing the risk of side effects. Reduced clearance of aprepitant and fosaprepitant is anticipated, enhancing clinical response and increasing risk of side effects; consider dose adjustment.
CYP2C9	*1/*1	Normal metabolism of celecoxib, piroxicam and flurbiprofen is anticipated (NSAIDs). Normal clearance of dronabinol and nabilone (synthetic cannabinoids which mimic THC) is anticipated.

Customer Genetic Profile

Biomarker	Value
ADRB2	AA
CYP2D6	*4/*4 (2N)
OPRM1	AA
CYP2B6	*1/*2
CYP2C19	*1/*2
CYP3A4	*1/*22
UGT1A1	*1/*80
UGT2B15	CA
CYP1A2	*1F/*1F
SLCO1B1	*1A/*15 or *1B/*5
CYP2C8	*1A/*1A
CYP2C9	*1/*1
CYP3A5	*3A/*3A
DPYD	*5/*6
F2	GG
F5	CC

Biomarker	Value
IFNL3	CC
TPMT	*1/*1
VKORC1	GG

GYIJAN072019|41001802063196|3967|2.1.2|179/179|CYP2B6:(*5,*7)|SLCO1B1:[*1A/*15=*1B/*5]
