# Supplementary material for: Implementation of Pharmacogenomics Testing in Daily Clinical Practice: Perspectives of Prescribers from Two Canadian Armed Forces Medical Clinics
Source: J Pers Med. 2025 Mar 4;15(3):101. doi: 10.3390/jpm15030101 (PMC11943113; doi:10.3390/jpm15030101)
Supplement: Supplementary file 1 [file jpm-15-00101-s001.zip › Table S1.pdf]

Supplemental Table S1: Interview guide with open-ended questions and probes

	Questions	Probes
Part 1 – Usefulness of Pharmacogenetics Testing	For what types of cases did you request pharmacogenetics testing?	Why did you think PGx test results would be useful for those cases?
	To what extent did your confidence in making prescribing decisions or adjustments for patients increase as a result of having access to pharmacogenetics test results?	If yes, in what ways did it reassure you?<br><br>If no, why not?
	In your experience to date, what has been the most significant value in having access to pharmacogenetics test results?	If perceived value, probe on:<br>1.Identifying patients at risk for adverse drug reactions<br>2.Greater insight into expected patient therapeutic response to recommended medication<br>3.Other<br>If no perceived value, why not?
	What, if any, important treatment impacts have you observed?	
	Can you briefly provide a case example of where having PGx test results has been valuable or where you have seen important treatment impacts?	
Part 2 – Ease of Use	How well were you able to integrate use of pharmacogenetics testing into your clinical workflow?	1. What could be improved to better integrate PGx testing into workflow?<br>2. How easily could you identify patients that could benefit from PGx testing?<br>3. Any concerns about having the time to introduce the PGx test to patients? Making the requisition?<br>4. Any concerns about access to PGx test results when needed or appropriate follow-up/scheduling to apply test

		results to the patient medication plan?<br>5. Any concerns about access to pharmacy consult, when needed?
	How helpful did you find the Pharmacogenetics Summary Report?	1. Did you refer to the PGx Summary report for most cases where you had ordered a PGx test? If not, why not?<br>2. What changes would you recommend in how the information is presented?<br>3. Could you easily find and understand the clinically relevant test results?<br>4. Did it give you the information you needed to apply test results to prescribing decisions? If not, what was missing?<br>5. Did you trust the recommendations? If no, why not?
Part 3 – Training and Support	How well did the training provided prepare you to use the pharmacogenetics testing service correctly?	1. Which of the training offerings did you participate in? (Workshop? On-line training modules? Personal use of PGx test?)<br>2. What was most helpful?<br>3. Is there anything that should be added or removed?<br>4. How could the training be improved?
